# Supplementary material for: Competition between hematopoietic stem and progenitor cells controls hematopoietic stem cell compartment size
Source: Nat Commun. 2022 Aug 8;13:4611. doi: 10.1038/s41467-022-32228-w (PMC9360400; doi:10.1038/s41467-022-32228-w)
Supplement: Supplementary file 1 — Supplementary Information [file 41467_2022_32228_MOESM1_ESM.docx]

Supplementary data for:

**Competition between hematopoietic stem and progenitor cells controls hematopoietic stem cell compartment size.**

Runfeng Miao1, Harim Chun2, Xing Feng1, Ana Cordeiro Gomes1#, Jungmin Choi2,3*, and João P. Pereira1*

1 Department of Immunobiology and Yale Stem Cell Center, Yale University School of Medicine, 300 Cedar Street, New Haven, CT 06519, USA.

2 BK21 Graduate Program, Department of Biomedical Sciences, Korea University College of Medicine, Seoul 02841, Republic of Korea (H.C., J.C.).

3 Department of Genetics, Yale University School of Medicine, 300 Cedar Street, New Haven, CT 06519, USA (J.C.)

# Current address: i3S – Instituto de Investigação e Inovação em Saúde, University of Porto, Porto, Portugal.

Correspondence: [joao.pereira@yale.edu](mailto:joao.pereira@yale.edu) and [jungmin.choi@yale.edu](mailto:jungmin.choi@yale.edu).

Lead contact: [joao.pereira@yale.edu](mailto:joao.pereira@yale.edu)

This supplementary information file contains:

Supplementary Figures 1-6.

**Supplementary Data 1.** Differentially expressed genes between bone marrow non-hematopoietic cell clusters from *Cxcr4*^+/+^ and *Cxcr4*^fl/fl^  Flk2-cre identified by scRNAseq. Related to Figure 4.

**Supplementary Data 2.** List of genes used to exclude cell contaminants. Related to Figure 4.

**Supplementary Data 3.** List of antibodies including concentrations and dilutions used in this study.


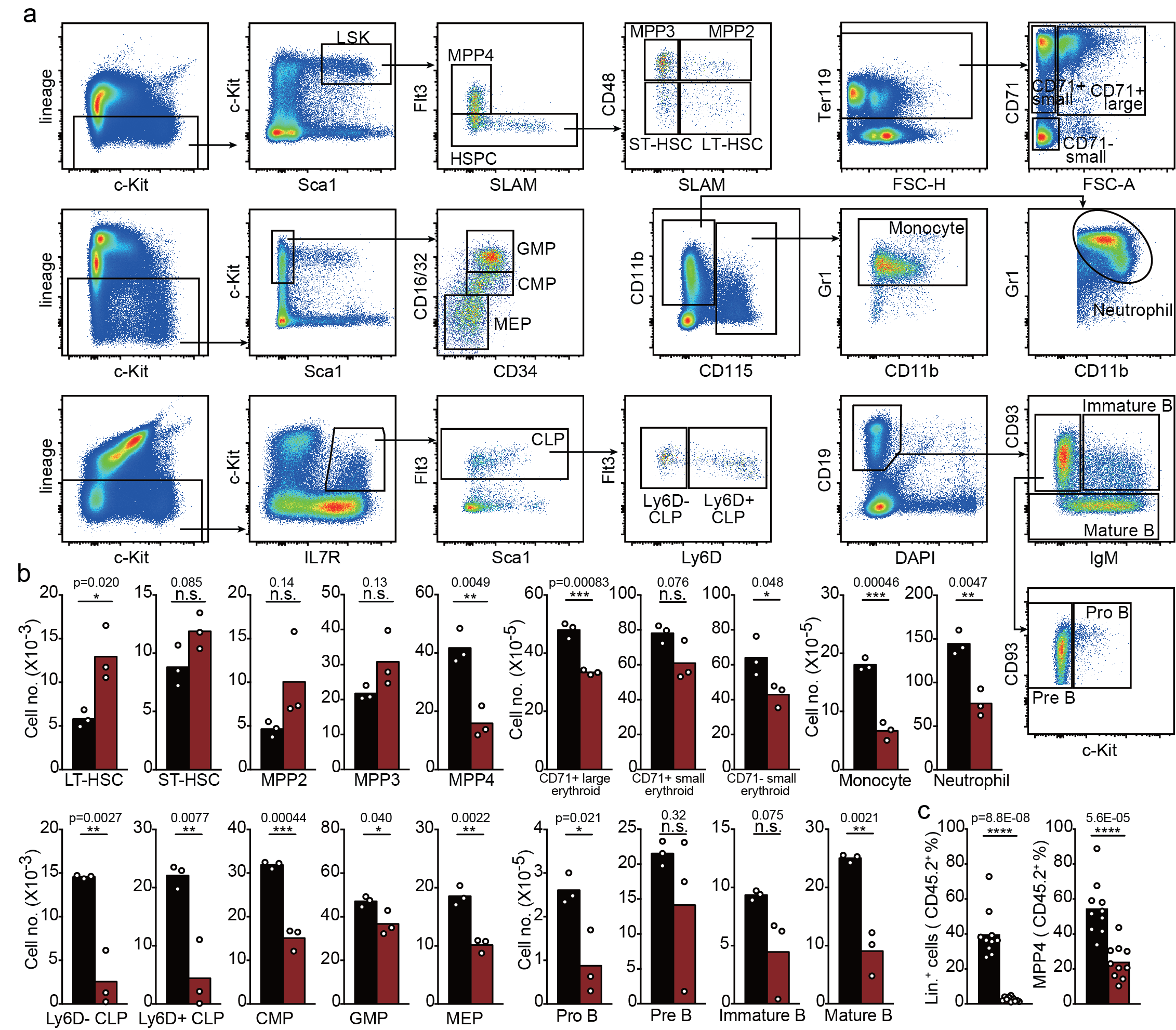


Supplementary **Figure 1. *Flk2-cre* is activated at the MPP stage.**

(A) Gating strategies for bone marrow cell populations. (B) Enumeration of LT-HSC, ST-HSC, MPP cell subsets, erythroid, myeloid, and lymphoid progenitors, B cell progenitors, and monocytes and neutrophils in femur and tibia of *Flk2-cre.Cxcr4^fl/+^* (black) and *Flk2-cre.Cxcr4^fl/fl^* (red) mice (n=3/group). (C) Chimerism of lineage positive cells and MPPs in bone marrow of lethally irradiated mice reconstituted with 50% CD45.2^+^ *Flk2-cre.Cxcr4^fl/+^* (black) or *Flk2-cre.Cxcr4^fl/fl^* (red) bone marrow cells mixed with 50% CD45.1^+^ wild-type bone marrow cells (n=10/group). Bars indicate average, circles depict individual mice. *, P < 0.05; **, P < 0.01, ***, P < 0.001, and ****, P < 0.0001 by unpaired two-sided Student’s *t* test. Source data are provided as a Source Data file.


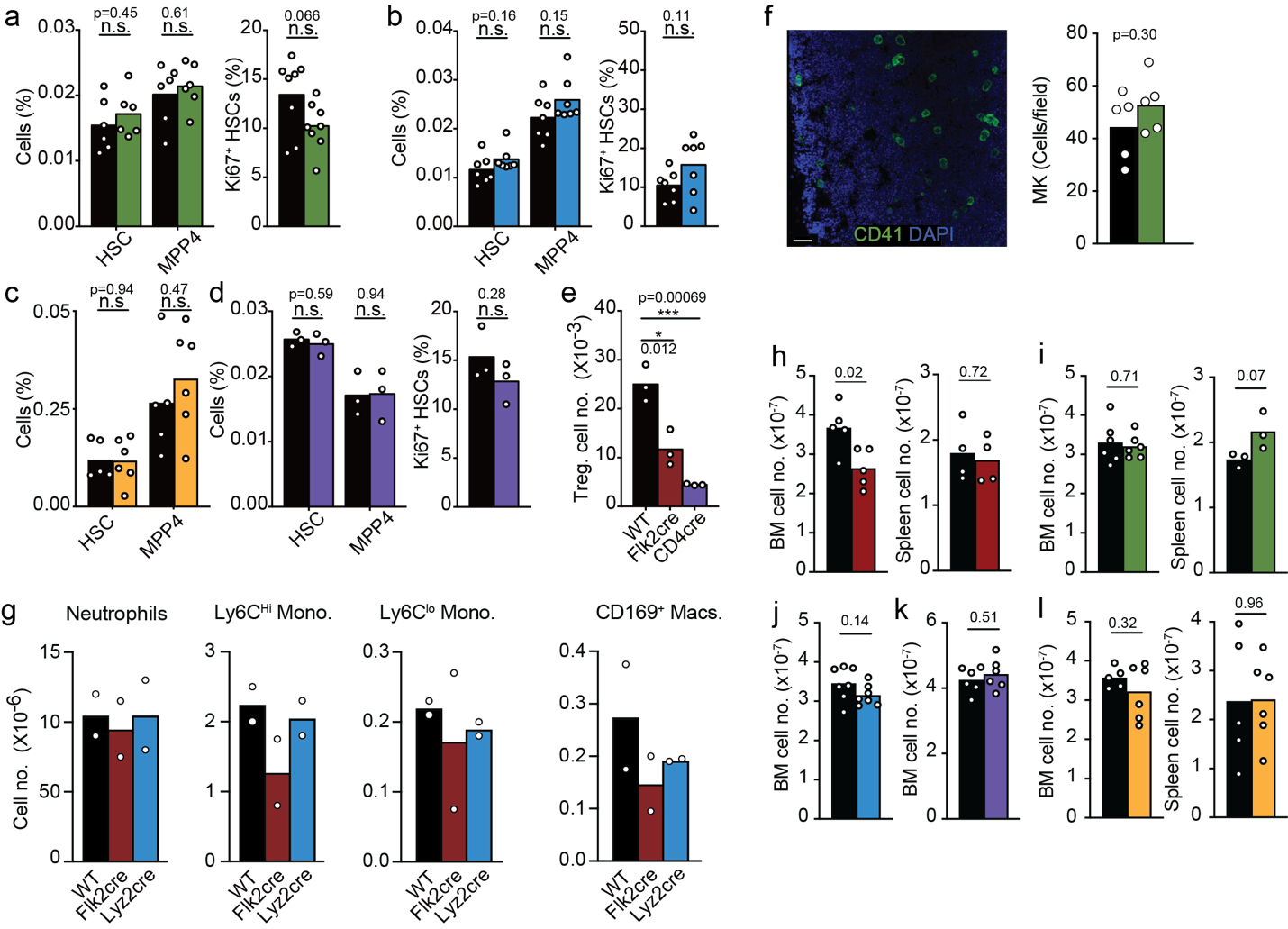


**Supplementary Figure 2. HSC frequency and quiescence in *Cxcr4* conditionally deficient mice.**

(A, B and D) HSC and MPP4 cell frequency; HSC cell cycle status. Control littermates (black), *Pf4-cre.Cxcr4^fl/fl^* (green, n=6/group), *Lyz2-cre.Cxcr4^fl/fl^* (blue, n=7/group) and *Cd4-cre.Cxcr4^fl/fl^* (purple, n=3/group) mice. Number of mice (circles) is indicated in each panel. (C) HSCs and MPP4 cell frequency in bone marrow of control (black, n=5/group) and *Il7ra-cre.Cxcr4^fl/fl^* (yellow, n=6/group) mice. (E) T regulatory (Tregs) cell number per femur and tibia: WT, wild-type; *Flk2*-cre*.Cxcr4^fl/fl^* (red), *Cd4*-cre*.Cxcr4^fl/fl^* (n=3/group). (F) Megakaryocyte numbers in *Pf4-cre.Cxcr4^fl/+^* (black) and *Pf4-cre.Cxcr4^fl/fl^* (green) mice (n=5/group). Megakaryocytes were enumerated by fluorescence microscopy analysis of 20 µm thick femur sections (left panel). Scale bar is 50µm. (G) Myeloid cell numbers in bone marrow of *Flk2*-cre*.Cxcr4^fl/fl^* (red), *Lyz2-cre.Cxcr4^fl/+^* (black) and *Lyz2-cre.Cxcr4^fl/fl^* (blue) mice (n=2/group). (H-L) Bone marrow and spleen cell numbers in Cxcr4 cKO mice. Number of mice (circles) is indicated in each panel. In panels H-L, control (black), *Flk2-cre.Cxcr4^fl/fl^* (red, bone marrow n=5/group, spleen n=4/group), *Pf4-cre.Cxcr4^fl/fl^* (green, bone marrow n=6/group, spleen n=3/group), *Lyz2-cre.Cxcr4^fl/fl^* (blue, n=7/group) and *Cd4-cre.Cxcr4^fl/fl^* (purple, n=6/group), and *Il7ra*^Cre/+^.*Cxcr4^fl/fl^* (yellow, CTR n=5, KO n=6 ) mice. Bars indicate average, circles depict individual mice. n.s., not significant, P > 0.05; *, P < 0.05 and *** P < 0.001 by unpaired two-sided Student’s *t* test. Source data are provided as a Source Data file.


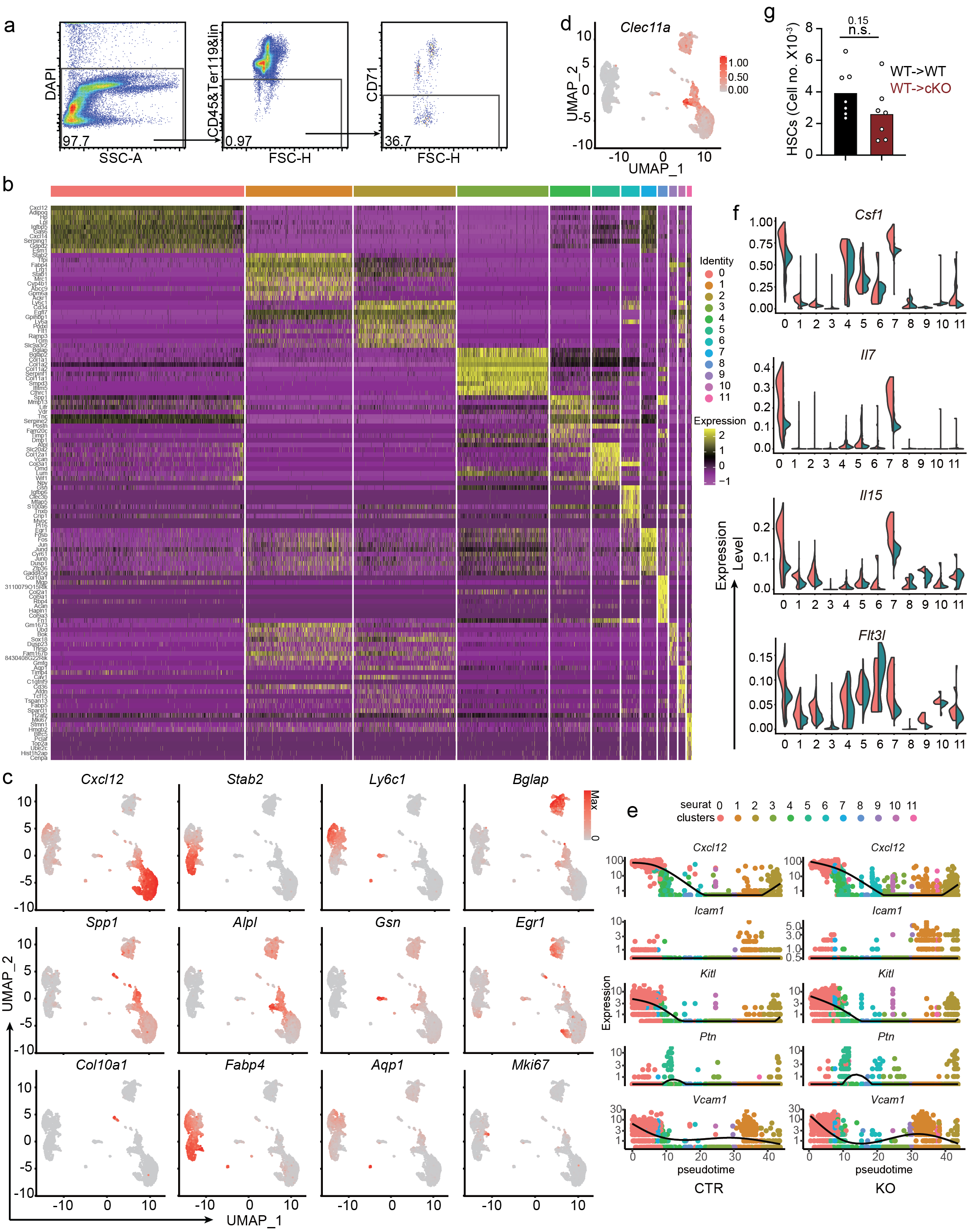


**Supplementary Figure 3. Niche cell heterogeneity and gene expression analyses.**

(A) Gating strategy used for sorting of bone marrow non-hematopoietic cells. (B) Cluster signature genes. Expression (row-wide Z-score of ln(TP10K+1)) of top differentially expressed genes (rows) across the cells (columns) in each cluster (color bar, top, indicates each cluster as in Fig. 4A). Genes are indicated on the left. (C) Expression levels of indicated cluster-defining genes overlaid on UMAP. (D) Osteolectin expression level overlaid on UMAP. (E) Expression level of the indicated genes in cell clusters of CTL and KO with respect to their pseudotime coordinates. Black lines depict LOESS regression fit of the normalized expression values of essential HSC regulators (*Kitl, Cxcl12, Ptn, Vcam1, Icam1*) in different clusters. (F) Violin plots representing expression levels of hematopoietic cell differentiation genes in CTL (red) and KO (blue) cell clusters. In panels E and F, CTL (*Flk2-cre.Cxcr4^fl/+^*) and KO (*Flk2-cre.Cxcr4^fl/fl^*). (G) HSC numbers in *Flk2-cre.Cxcr4^fl/+^* (CTR, black) and *Flk2-cre.Cxcr4^fl/fl^* mice (cKO, red) mice lethally irradiated and reconstituted with wild-type bone marrow cells (n=7/group). n.s., not significant, unpaired two-sided Student’s *t* test.


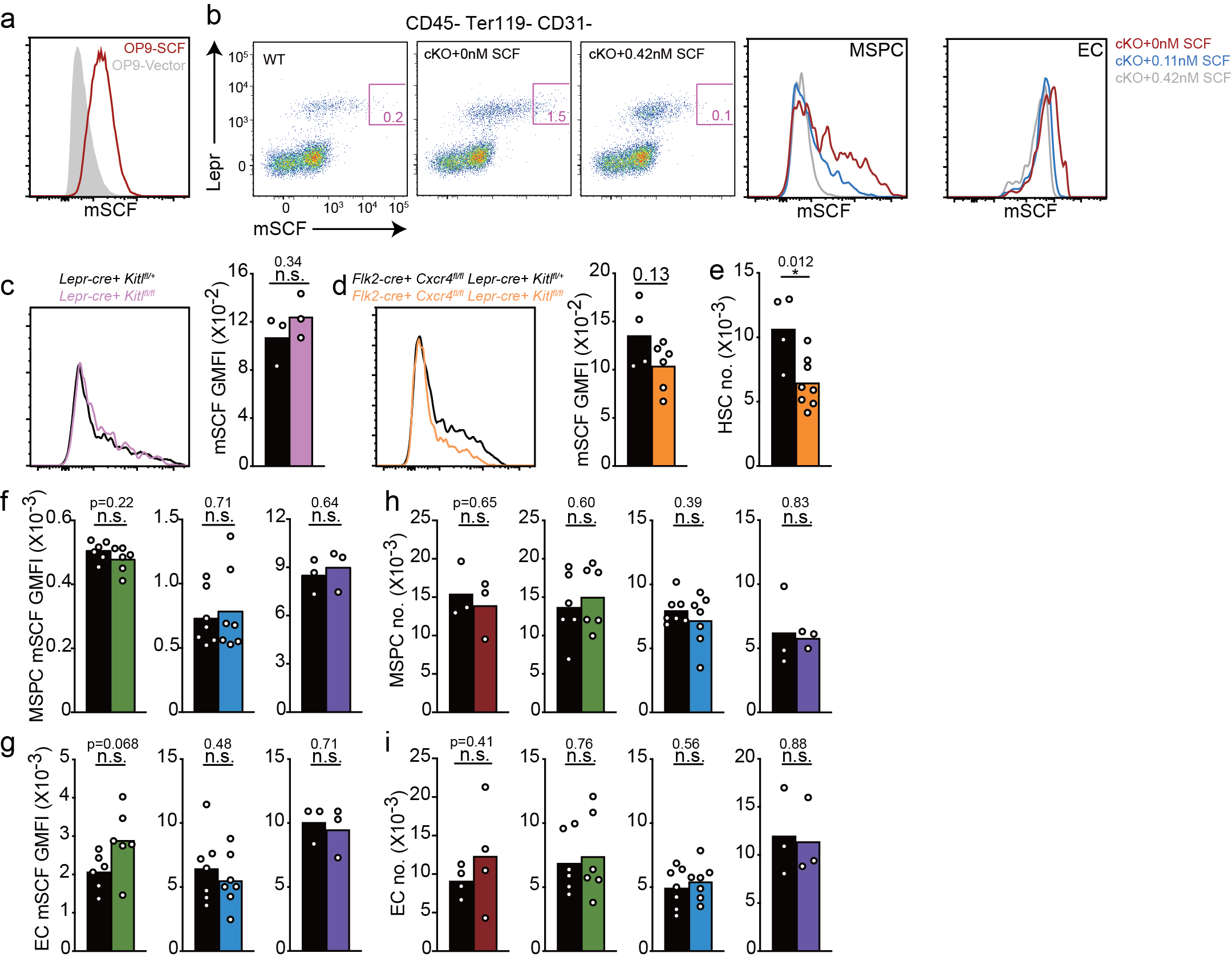


**Supplementary Figure 4. mSCF measurements and staining specificity.**

(A) Histogram overlay of mSCF staining in OP9 cells transduced with Empty-Vector pMSCV (gray) or with *Kitl*-expressing pMSCV (red) (B) histograms in gated LEPR^+^ MSPCs (left) and ECs (right) of secondary antibody (gray), or of anti-SCF pre-incubated for 30 minutes at room temperature with 0 nM (red), 0.105 nM (blue), or 0.421 nM (dark gray) mouse recombinant SCF. (C) mSCF histogram overlay of LEPR^+^ MSPCs isolated from *Lepr*-cre^+^ *Kitl^fl/+^* (black) and *Lepr*-cre^+^ *Kitl^fl/fl^* (pink) mice (n=3/group). (D) mSCF histogram overlay of LEPR^+^ MSPCs isolated from *Lepr*-cre^+^ *Kitl^fl/+^* *Flk2-cre^+^ Cxcr4^fl/fl^* (black, n=4) and *Lepr*-cre^+^ *Kitl^fl/fl^* *Flk2-cre^+^ Cxcr4^fl/fl^* (orange, n=6) mice. (E) HSC numbers in bone marrow of *Lepr*-cre^+^ *Kitl^fl/+^* *Flk2-cre^+^ Cxcr4^fl/fl^* (black, n=4) and *Lepr*-cre^+^ *Kitl^fl/fl^* *Flk2-cre^+^ Cxcr4^fl/fl^* (orange, n=8) mice. (F and G) mSCF expression on LEPR^+^ MSPCs (F) and ECs (G); (H and I) LEPR^+^ MSPC and ECs cell numbers per femur and tibia. (F-I) control (black), *Flk2-cre.Cxcr4^fl/fl^* (red, n=3/group), *Pf4-cre.Cxcr4^fl/fl^* (green, n=6/group), *Lyz2-cre.Cxcr4^fl/fl^* (blue, n=7/group) and *Cd4-cre.Cxcr4^fl/fl^* (purple, n=3/group) mice. Bars indicate average, circles depict individual mice. n.s., not significant. P > 0.05; *, P < 0.05 by unpaired two-sided Student’s *t* test. Source data are provided as a Source Data file.


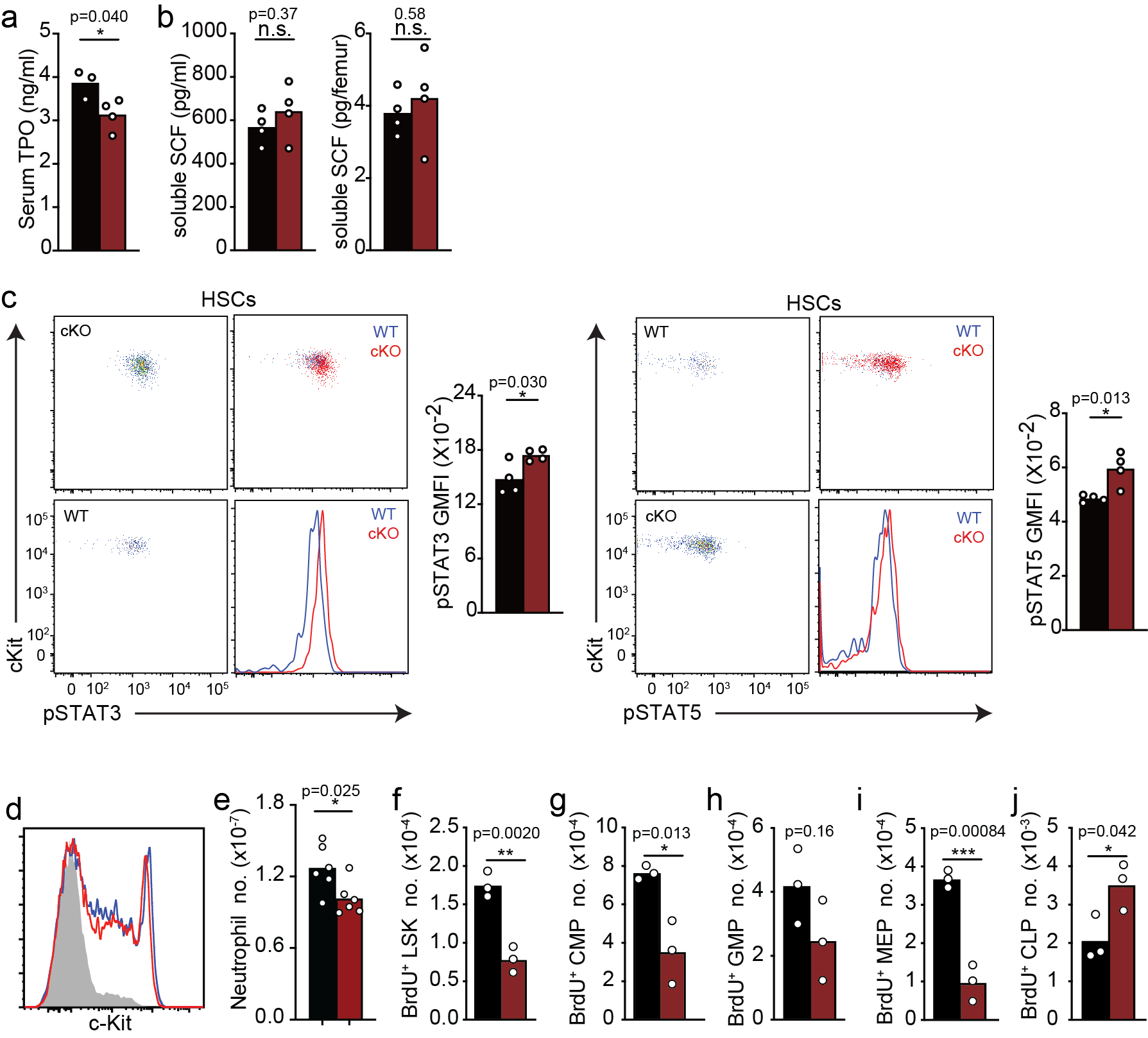


**Supplementary Figure 5. Cytokine measurements, cKit signaling, and cKit blocking saturation.**

(A) Serum Thrombopoietin (CTR n=3, cKO n=4). (B) Soluble SCF in bone marrow interstitial fluid (n=4/group). (A and B) *Flk2-cre.Cxcr4^fl/+^* (black) and *Flk2-cre.Cxcr4^fl/fl^* (red) mice. (C) pSTAT3 and pSTAT5 staining in HSCs incubated with 600 ng/ml mouse recombinant SCF (red) or medium (black). Numbers indicate GMFI (n=4/group). (D) cKit staining of bone marrow Lineage^-^ Sca-1^+^ cells in wild-type mice at day 3 after treatment with ACK2 (Rat-anti-mouse cKit) antibody or isotype control (200µg/mouse). Secondary antibody (anti-Rat IgG) staining of cells from isotype control treated mouse (gray); secondary antibody staining of cells from ACK2 antibody treated mouse (red), ACK2 and secondary antibody staining of cells from ACK2 antibody treated mouse (blue). (E) Neutrophil cell number in the bone marrow of control or ACK2-treated mice. Data in all panels are representative of at least two independent experiments (n=6/group). (F-J) BrdU+ LSK, CMP, GMP, MEP and CLP cell numbers in bone marrow of mice treated with ACK2 (200µg/mouse) for 24h (n=3/group). Bars indicate average, circles depict individual mice. *, P < 0.05 by unpaired two-sided Student’s *t* test. Source data are provided as a Source Data file.


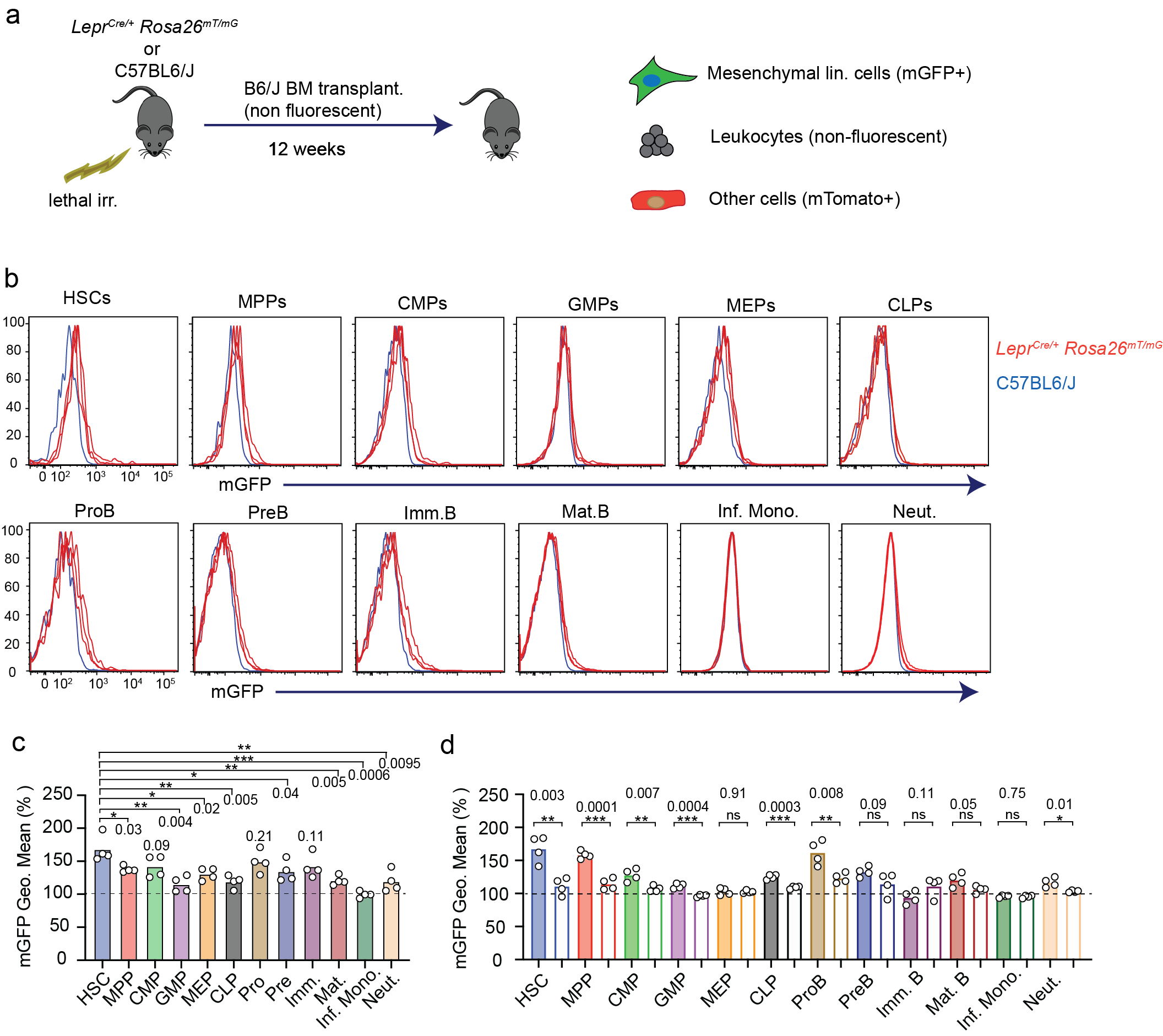


**Supplementary Figure 6. Hematopoietic cell acquisition of MSPC membrane processes (trogocytosis) in vivo.** (A) experiment design: *Lepr^Cre/+^ Rosa26 ^mT/mG^* mice were lethally irradiated and reconstituted with CD45.1+ BM cells (C57BL6/J). The level of mGFP fluorescence was determined on hematopoietic cell subsets 12 weeks after reconstitution and compared to background fluorescence detected in BM cells from C57BL6/J mice. (B) Histogram of mGFP fluorescence in the indicated hematopoietic cell subsets. (C) Comparison of mGFP fluorescence intensity in hematopoietic cell subsets displayed as percentage over background fluorescence measured in BM cell subsets from C57BL6/J mice (n=4). (D) In vivo versus ex vivo acquisition of mGFP fluorescence by hematopoietic cells. Filled bars indicate mGFP fluorescence acquired in vivo. Open bars show ex vivo acquisition of mGFP fluorescence by CD45.2+ C57BL6/J BM cells mixed ex vivo with BM cells from *Lepr^Cre/+^ Rosa26 ^mT/mG^* chimeric mice (n=4/group). In panels C and D, circles depict individual mice. Data in all panels are representative of 2 individual experiments. n.s., not significant, P > 0.05; *, P < 0.05, ** P < 0.01 and *** P < 0.001 by unpaired two-sided Student’s *t* test. Source data are provided as a Source Data file.
