# Supplementary material for: Competition between hematopoietic stem and progenitor cells controls hematopoietic stem cell compartment size
Source: Nat Commun. 2022 Aug 8;13:4611. doi: 10.1038/s41467-022-32228-w (PMC9360400; doi:10.1038/s41467-022-32228-w)
Supplement: Supplementary file 3 — Description of Additional Supplementary Files [file 41467_2022_32228_MOESM3_ESM.pdf]

Description of Additional Supplementary Files

Supplementary Data 1. Differentially expressed genes between bone marrow non-hematopoietic cell clusters from *Cxcr4*$^{+/+}$ and *Cxcr4*$^{fl/fl}$ Flk2-cre identified by scRNAseq. Related to Figure 4.

Supplementary Data 2. List of genes used to exclude cell contaminants. Related to Figure 4.

Supplementary Data 3. List of antibodies including concentrations and dilutions used in this study.
