# Supplementary material for: Competition between hematopoietic stem and progenitor cells controls hematopoietic stem cell compartment size
Source: Nat Commun. 2022 Aug 8;13:4611. doi: 10.1038/s41467-022-32228-w (PMC9360400; doi:10.1038/s41467-022-32228-w)
Supplement: Supplementary file 7 — Reporting Summary [file 41467_2022_32228_MOESM7_ESM.pdf]

nature portfolio

Corresponding author(s): Joao P. Pereira; Jungmin Choi

Last updated by author(s): Jun 6, 2022

Reporting Summary

Nature Portfolio wishes to improve the reproducibility of the work that we publish. This form provides structure for consistency and transparency in reporting. For further information on Nature Portfolio policies, see our Editorial Policies and the Editorial Policy Checklist.

Statistics

For all statistical analyses, confirm that the following items are present in the figure legend, table legend, main text, or Methods section.

n/a	Confirmed	
[ ]	[x]	The exact sample size (*n*) for each experimental group/condition, given as a discrete number and unit of measurement
[x]	[ ]	A statement on whether measurements were taken from distinct samples or whether the same sample was measured repeatedly
[ ]	[x]	The statistical test(s) used AND whether they are one- or two-sided<br>*Only common tests should be described solely by name; describe more complex techniques in the Methods section.*
[x]	[ ]	A description of all covariates tested
[x]	[ ]	A description of any assumptions or corrections, such as tests of normality and adjustment for multiple comparisons
[ ]	[x]	A full description of the statistical parameters including central tendency (e.g. means) or other basic estimates (e.g. regression coefficient) AND variation (e.g. standard deviation) or associated estimates of uncertainty (e.g. confidence intervals)
[ ]	[x]	For null hypothesis testing, the test statistic (e.g. *F*, *t*, *r*) with confidence intervals, effect sizes, degrees of freedom and *P* value noted<br>*Give P values as exact values whenever suitable.*
[x]	[ ]	For Bayesian analysis, information on the choice of priors and Markov chain Monte Carlo settings
[x]	[ ]	For hierarchical and complex designs, identification of the appropriate level for tests and full reporting of outcomes
[x]	[ ]	Estimates of effect sizes (e.g. Cohen's *d*, Pearson's *r*), indicating how they were calculated

Our web collection on statistics for biologists contains articles on many of the points above.

Software and code

Policy information about availability of computer code

Data collection	Data collection methods used in this study were thoroughly described in the Methods section. Briefly, BD FACSDiva v8.0 and v9.0, 10x Genomic Cell Ranger 4.0.0.
Data analysis	All data analysis methods used in this study were thoroughly described in the Methods section. Briefly, FlowJo Software (10.5.3; Tree Star), HISAT2, SAMtools, Seurat R Package 4.0, SCTransform, HTSeq-count, DESeq2.

For manuscripts utilizing custom algorithms or software that are central to the research but not yet described in published literature, software must be made available to editors and reviewers. We strongly encourage code deposition in a community repository (e.g. GitHub). See the Nature Portfolio guidelines for submitting code & software for further information.

Data

Policy information about availability of data

All manuscripts must include a data availability statement. This statement should provide the following information, where applicable:

- Accession codes, unique identifiers, or web links for publicly available datasets
- A description of any restrictions on data availability
- For clinical datasets or third party data, please ensure that the statement adheres to our policy

All bulk RNA-seq and scRNA-seq data were deposited in the Gene Expression Omnibus (GEO) under the accession number GSE171015. The following databases were also used: Mus musculus GRCm38 (mm10)

Field-specific reporting

Please select the one below that is the best fit for your research. If you are not sure, read the appropriate sections before making your selection.

☒ Life sciences ☐ Behavioural & social sciences ☐ Ecological, evolutionary & environmental sciences

For a reference copy of the document with all sections, see nature.com/documents/nr-reporting-summary-flat.pdf

Life sciences study design

All studies must disclose on these points even when the disclosure is negative.

Sample size	Sample sizes were based on previous studies (Pereira et al. Nat. Immunol 2009; Pereira et al. Nature, 2009; Beck et al. JEM 2014; Nevius et al. JEM 2015; Gomes et al. Immunity 2016; Fistonich et al. JEM 2018). Briefly, we used the minimum number of mice that allowed revealing statistical significance (Student's t test) between groups therefore ensuring that a balance between statistical robustness and animal welfare was achieved.
Data exclusions	No data were excluded from analyses.
Replication	All experiments described in this study were independently reproduced at least twice, with the exception of RNA sequencing studies that were performed once due to costs. For the most part, in vivo studies were designed such that each hypothesis was tested by independent approaches (e.g. comparing conditional gene targeting results with in vivo antibody blocking experiments).
Randomization	No specific method was used. All in vivo studies used littermates as controls; when cre transgenic mice result in heterozygous mutation of an immunologically important gene (e.g. Il7Ra) cre+ mice were used in all experimental groups.
Blinding	Experiments were not blinded because the measurements were quantitative (e.g. total cell numbers, abundance of a certain protein, etc).

Reporting for specific materials, systems and methods

We require information from authors about some types of materials, experimental systems and methods used in many studies. Here, indicate whether each material, system or method listed is relevant to your study. If you are not sure if a list item applies to your research, read the appropriate section before selecting a response.

Materials & experimental systems

n/a	Involved in the study
☐	☒ Antibodies
☒	☐ Eukaryotic cell lines
☒	☐ Palaeontology and archaeology
☐	☒ Animals and other organisms
☒	☐ Human research participants
☒	☐ Clinical data
☒	☐ Dual use research of concern

Methods

n/a	Involved in the study
☒	☐ ChIP-seq
☐	☒ Flow cytometry
☒	☐ MRI-based neuroimaging

Antibodies

Antibodies used

Target Clone Cat. No.
SCF 40215 AF-455-NA
SCF 40215 BAF455
Brdu 51-23614L
Ki67 SolA15 50-5698-82
pSTAT3 13A3-1 651009
pSTAT3 47/Stat5 (pY694) 562984
cKIT 2B8 105835
SCA1 D7 108114
FLT3 A2F10 135310
CD150 TC15-12F12.2 115904
CD19 6D5 115521
B220 RA3-6B2 103206
CD3e 145-2C11 100306
CD4 RM4-5 100510
Gr1 RB6-8C5 108406
NK1.1 PK136 108706
Ter119 TER-119 116206

CD11b M1/70 101206
CD11c N418 117306
CD41 MWReg30 133904
CD48 HM48-1 103404
CD25 PC61 102017
CD45.1 A20 110716
CD45.2 104 109824
Ter119 TER-119 116223
CD31 390 102410
CD144 BV13 138006
PDGFRa APA5 135906
PDGFRb APB5 136007
LEPR BAF497
CD19 6D5 115504
B220 RA3-6B2 103204
CD3e 145-2C11 100304
CD4 RM4-5 100508
Gr1 RB6-8C5 108404
NK1.1 PK136 108704
Ter119 TER-119 13-5921-82
CD11b M1/70 101204
CD11c N418 117304

Validation

https://www.rndsystems.com/products/mouse-scf-c-kit-ligand-antibody_af-455-na
https://www.rndsystems.com/products/mouse-scf-c-kit-ligand-biotinylated-antibody_baf455
https://www.bdbiosciences.com/en-eu/products/reagents/flow-cytometry-reagents/research-reagents/panels-multicolor-cocktails-ruo/fitc-brdu-flow-kit.559619
https://www.thermofisher.com/antibody/product/Ki-67-Antibody-clone-SolA15-Monoclonal/50-5698-82
https://www.biolegend.com/de-at/products/brilliant-violet-421-anti-stat3-phospho-tyr705-antibody-13030
https://www.bdbiosciences.com/en-fi/products/reagents/flow-cytometry-reagents/research-reagents/single-color-antibodies-ruo/BV421-Mouse-Anti-Stat5-(pY694).562984
https://www.biolegend.com/nl-be/products/brilliant-violet-711-anti-mouse-cd117-c-kit-antibody-12049
https://www.biolegend.com/it-it/products/pe-cyanine7-anti-mouse-ly-6a-e-sca-1-antibody-3137
https://www.biolegend.com/it-it/products/apc-anti-mouse-cd135-antibody-6284
https://www.biolegend.com/fr-fr/products/pe-anti-mouse-cd150-slam-antibody-1369
https://www.biolegend.com/fr-ch/products/alexa-fluor-488-anti-mouse-cd19-antibody-2704
https://www.biolegend.com/fr-lu/products/fitc-anti-mouse-human-cd45r-b220-antibody-445
https://www.biolegend.com/it-it/products/fitc-anti-mouse-cd3epsilon-antibody-23
https://www.biolegend.com/fr-lu/products/fitc-anti-mouse-cd4-antibody-480
https://www.biolegend.com/fr-ch/products/fitc-anti-mouse-ly-6g-ly-6c-gr-1-antibody-458
https://www.biolegend.com/ja-jp/products/fitc-anti-mouse-nk-1-1-antibody-429
https://www.biolegend.com/it-it/products/fitc-anti-mouse-ter-119-erythroid-cells-antibody-1865
https://www.biolegend.com/de-at/products/fitc-anti-mouse-human-cd11b-antibody-347
https://www.biolegend.com/en-us/search-results/fitc-anti-mouse-cd11c-antibody-1815?GroupID=BLG11937
https://www.biolegend.com/de-de/products/fitc-anti-mouse-cd41-antibody-5896
https://www.biolegend.com/nl-be/products/fitc-anti-mouse-cd48-antibody-291
https://www.biolegend.com/nl-be/products/alexa-fluor-488-anti-mouse-cd25-antibody-2706
https://www.biolegend.com/de-de/products/apc-cyanine7-anti-mouse-cd45-1-antibody-2320
https://www.biolegend.com/fr-lu/products/apc-cyanine7-anti-mouse-cd45-2-antibody-3906
https://www.biolegend.com/fr-fr/products/apc-cyanine7-anti-mouse-ter-119-erythroid-cells-antibody-3905
https://www.biolegend.com/it-it/products/apc-anti-mouse-cd31-antibody-118
https://www.biolegend.com/nl-be/products/alexa-fluor-647-anti-mouse-cd144-ve-cadherin-antibody-6569
https://www.biolegend.com/nl-be/products/pe-anti-mouse-cd140a-antibody-6253
https://www.biolegend.com/it-it/products/apc-anti-mouse-cd140b-antibody-6441
https://www.rndsystems.com/products/mouse-leptin-r-biotinylated-antibody_baf497
https://www.biolegend.com/fr-ch/products/biotin-anti-mouse-cd19-antibody-1527
https://www.biolegend.com/de-de/products/biotin-anti-mouse-human-cd45r-b220-antibody-444
https://www.biolegend.com/it-it/products/biotin-anti-mouse-cd3epsilon-antibody-22
https://www.biolegend.com/it-it/products/biotin-anti-mouse-cd4-antibody-479
https://www.biolegend.com/ja-jp/products/biotin-anti-mouse-ly-6g-ly-6c-gr-1-antibody-457
https://www.biolegend.com/de-de/products/biotin-anti-mouse-nk-1-1-antibody-428
https://www.biolegend.com/it-it/products/fitc-anti-mouse-ter-119-erythroid-cells-antibody-1865
https://www.biolegend.com/fr-fr/products/biotin-anti-mouse-human-cd11b-antibody-346
https://www.biolegend.com/en-us/search-results/biotin-anti-mouse-cd11c-antibody-1814?GroupID=BLG11937

Animals and other organisms

Policy information about studies involving animals; ARRIVE guidelines recommended for reporting animal research

Laboratory animals	C57BL/6NCR of both sexes (strain code 556) (CD45.2+) and B6-Ly5.1/Cr (stain code 564) of both sexes (CD45.1+) were purchased from Charles River Laboratories. Male a female Rosa26mTmG, Pf4-cre, Kitlfl/fl, Lepr-cre, and Kitl GFP/+ mice were from The Jackson Laboratories. Cxcr4fl/fl, Lyz2-cre, Il7ra-cre, CD4-cre, and Rosa26tdtomato/+ mice of both sexes were from internal colonies. Flk2-cre male mice were a gift from Dr. E. Camilla Forsberg (University of California, Santa Cruz). Although Flk2-cre transgene is inserted into Y-chromosome, our previous work showed the hematopoietic cell composition in bone marrow and secondary lymphoid organs of Flk2-cre transgenic mice is indistinguishable from their littermate controls. All mice analyzed were 8-20 week old. Mice were housed in a specific pathogen-free (SPF) animal research facility, under 12h light/dark cycles, at 22C.
Wild animals	This study did not use wild animals.
Field-collected samples	No field collected samples were used in the study.
Ethics oversight	All animals were used according to the protocol approved by the Yale University Institutional Animal Care and Use Committee.

Note that full information on the approval of the study protocol must also be provided in the manuscript.

Flow Cytometry

Plots

Confirm that:

- [x] The axis labels state the marker and fluorochrome used (e.g. CD4-FITC).
- [x] The axis scales are clearly visible. Include numbers along axes only for bottom left plot of group (a 'group' is an analysis of identical markers).
- [x] All plots are contour plots with outliers or pseudocolor plots.
- [x] A numerical value for number of cells or percentage (with statistics) is provided.

Methodology

Sample preparation	Bone marrow stromal cells were isolated as follows The bones after flushing were chopped into small pieces and digested with HBSS supplemented with 2% of FBS, 1% Penicillin/Streptomycin, 1% L-glutamine, 1% HEPES, and 200 U/mL Collagenase IV (Worthington Biochemical Corporation) at 37°C for 45 min under agitation (120rpm). Cells were then filtered through 100 µm cell strainers before combined with digested bone marrow stromal cells.
Instrument	BD LSRII
Software	HISAT2, SAMtools, HTSeq-count, DESeq2, 10x Genomic Cell Ranger 4.0.0, Seurat R Package 4.0, SCTransform, Seurat, MiloR, FACSDiva v.8.02, FlowJo v10
Cell population abundance	MSPCs sorted for bulk RNA sequencing were double sorted with final purity checked by FACS at >98%.
Gating strategy	Cell gatings are displayed in main and supplementary datasets.

- [x] Tick this box to confirm that a figure exemplifying the gating strategy is provided in the Supplementary Information.
